# Cell Invasion by *Neisseria meningitidis* Requires a Functional Interplay between the Focal Adhesion Kinase, Src and Cortactin

**DOI:** 10.1371/journal.pone.0039613

**Published:** 2012-06-29

**Authors:** Heiko Slanina, Sabrina Hebling, Christoph R. Hauck, Alexandra Schubert-Unkmeir

**Affiliations:** 1 Institute of Hygiene and Microbiology, University of Würzburg, Würzburg, Germany; 2 Department of Cell Biology, University of Konstanz, Konstanz, Germany; University of Birmingham, United Kingdom

## Abstract

Entry of *Neisseria meningitidis* (the meningococcus) into human brain microvascular endothelial cells (HBMEC) is mediated by fibronectin or vitronectin bound to the surface protein Opc forming a bridge to the respective integrins. This interaction leads to cytoskeletal rearrangement and uptake of meningococci. In this study, we determined that the focal adhesion kinase (FAK), which directly associates with integrins, is involved in integrin-mediated internalization of *N. meningitidis* in HBMEC. Inhibition of FAK activity by the specific FAK inhibitor PF 573882 reduced Opc-mediated invasion of HBMEC more than 90%. Moreover, overexpression of FAK mutants that were either impaired in the kinase activity or were not capable of autophosphorylation or overexpression of the dominant-negative version of FAK (FRNK) blocked integrin-mediated internalization of *N. meningitidis*. Importantly, FAK-deficient fibroblasts were significantly less invaded by *N. meningitidis*. Furthermore, *N. meningitidis* induced tyrosine phosphorylation of several host proteins including the FAK/Src complex substrate cortactin. Inhibition of cortactin expression by siRNA silencing and mutation of critical amino acid residues within cortactin, that encompass Arp2/3 association and dynamin binding, significantly reduced meningococcal invasion into eukaryotic cells suggesting that both domains are critical for efficient uptake of *N. meningitidis* into eukaryotic cells. Together, these results indicate that *N. meningitidis* exploits the integrin signal pathway for its entry and that FAK mediates the transfer of signals from activated integrins to the cytoskeleton. A cooperative interplay between FAK, Src and cortactin then enables endocytosis of *N. meningitidis* into host cells.

## Introduction

*Neisseria meningitidis* is a commensal organism found frequently in the respiratory tract of healthy individuals. In rare cases, *N. meningitidis* can cause severe septicaemia and/or meningitis. *N. meningitidis* is able to attach and invade a variety of cell types using several microbial structures and proteins, including type IV pili (TfP), the major outer membrane adhesin proteins Opa and Opc and the newly identified minor adhesion or adhesion-like proteins [1]–[5]. The primary meningococcal invasins that facilitate bacterial uptake by endothelial cells are Opa and Opc.

*N. meningitidis* Opc is encoded by a single gene (*opcA*) and is antigenically relatively invariant [6], [7]. The *opcA* gene is widespread in epidemic and endemic *N. meningitidis*, but certain *N. meningitidis* clonal lineages, such as ST11 complex meningococci, lack *opcA* and tend to cause severe sepsis instead of meningitis [8]–[10]. Furthermore Opc expression is controlled at the transcriptional level and is determined by a poly C tract in the promoter region of the gene that influences the efficacy of RNA polymerase binding [11]. Although not universally present in the *N. meningitidis* Opc is expressed in numerous clinical isolates and retained by several meningococcal hypervirulent clonal lineages.

It has been shown that Opc confers the property of cellular invasion, especially of endothelial cells [5], [12], [13], through a tight association of the bacteria with extracellular matrix (ECM) proteins, such as vitronectin and fibronectin [8], [14], [15]. Both, vitronectin and fibronectin, are also abundant in human serum [16], [17] and Opc interaction with these serum factors leads to binding to endothelial αvβ3 integrin (the vitronectin receptor) and α5β1-integrin (the fibronectin receptor) [5], [8], [14]. This interaction promotes the uptake of *N. meningitidis* by the endothelial cell, a process, which requires rearrangement of the cytoskeleton [18].

Integrins are relatively large heterodimeric transmembrane proteins composed of a α and β subunit [19]. There are over 20 different members of the integrin family, many of which recognize an arginine, glycine, aspartic acid (RGD) sequence in host ECM proteins. Interactions of integrins with these ligands serve a number of important host cell functions including cell attachment, migration, growth, and differentiation. Besides *N. meningitidis*, several microbial pathogens are able to bind to integrins of mammalian cells either directly or indirectly, via ECM proteins and utilize these integrins as receptor for their uptake into host cells [20]–[23]. The mechanism, however, how binding of bacteria to integrins is translated into orchestrating the cellular cytoskeleton is not fully understood.

Upon cell adhesion, clustering of integrins to immobilised ECM proteins triggers the assembly of a multiprotein complex, focal adhesion, at the cytoplasmatic face of integrins [24]–[27]. Proteins enriched at focal adhesions include structural components such as vinculin, talin, paxillin, tensin, α-actinin, as well as signaling molecules such as the focal adhesion kinase (FAK), phosphatidylinositol phosphate kinase type1γ, the integrin linked kinase as a scaffold protein and Src family protein tyrosine kinases (PTKs), that together orchestrate the dynamic linkage between integrins and the actin cytoskeleton [26], [28]–[30]. We have recently shown that Src family PTKs are responsible for the transfer of signals from activated integrins to the cytoskeleton and thus mediate the endocytosis of *N. meningitidis* into human brain microvascular endothelial cells (HBMEC) [18], [31]. Since Src PTKs function in concert with the non-receptor PTK FAK, we hypothesized that FAK plays a major role in the invasion process.

The PTK FAK is one of the key enzymes highly activated upon integrin-mediated cell activation [32]. FAK, a widely expressed nonreceptor PTK, is a 125-kDA protein that contains a central kinase domain flanked by an amino-terminal and a carboxy-terminal domain. The amino-terminal domain contains an autophosphorylation site (Tyr397), which serves as a docking site for the Src homology 2 (SH2)-domain of Src-family PTKs. The complex formed by FAK and c-Src leads to Src-mediate phosphorylation of FAK at multiple sites in the kinase and carboxy-terminal domain [33]. The carboxy-terminal domain furthermore contains a region required for localization of FAK to focal adhesions (FAT (focal adhesion targeting) region) and binding sites for the cytoskeletal proteins paxillin and talin, which in part facilitate the recruitment of FAK to the cytoplasmatic tail of β-integrins.

Human brain cells express several alternative FAK splice variants that are able to regulate FAK phosphorylation. As such FRNK, the FAK-related non-kinase is expressed as an independent transcript encompassing the FAK c-terminal domain, but lacks kinase activity and the autophosphorylation site at Tyr397. Overexpression of FRNK has been demonstrated to inhibit endogenous integrin-mediated FAK activation by displacement and by competitive binding to integrin-associated proteins such as paxillin [32]. Thus, FAK is a tightly controlled factor that functions as a receptor-proximal regulator of cell shape, adhesion and motility [34].

In this study, we analyzed the role of FAK in the invasion process of *N. meningitidis* into eukaryotic cells in detail. Using a variety of approaches including pharmacological inhibitors, overexpression of the wild-type form of FAK, dominant-negative or genetically modified mutant forms, we demonstrate the crucial role of FAK in meningococcal entry. We show that both phosphorylation of FAK at Tyr397 and the kinase activity are critical in mediating uptake of *N. meningitidis* into the eukaryotic cell. In addition, FAK-deficient fibroblasts are significantly less invaded by meningococci. Meningococcal binding to integrins also induces enhanced tyrosine phosphorylation of the FAK/Src complex substrate cortactin. Cortactin is an actin-binding protein and a key regulator of actin rearrangement in response to tyrosine kinase signaling. Mutation of critical cortactin amino acids (W525K in the SH3 domain that interacts with dynamin and W22A point mutation in the N-terminal acidic domain (NTA) that activates the Arp2/3 actin polymerization machinery [35]) or siRNA-mediated gene silencing of cortactin blocked *N. meningitidis* invasion, supporting the hypothesis that both domains are critical for efficient uptake of *N. meningitidis* into host cells. Together, these data provide evidence that Src, cortactin and FAK are required for Opc-mediated cell invasion by *N. meningitidis*.

## Results

### Actin-cytoskeleton Dynamic is Essential for Integrin-mediated Internalization of *N. meningitidis*

**Figure 1:**
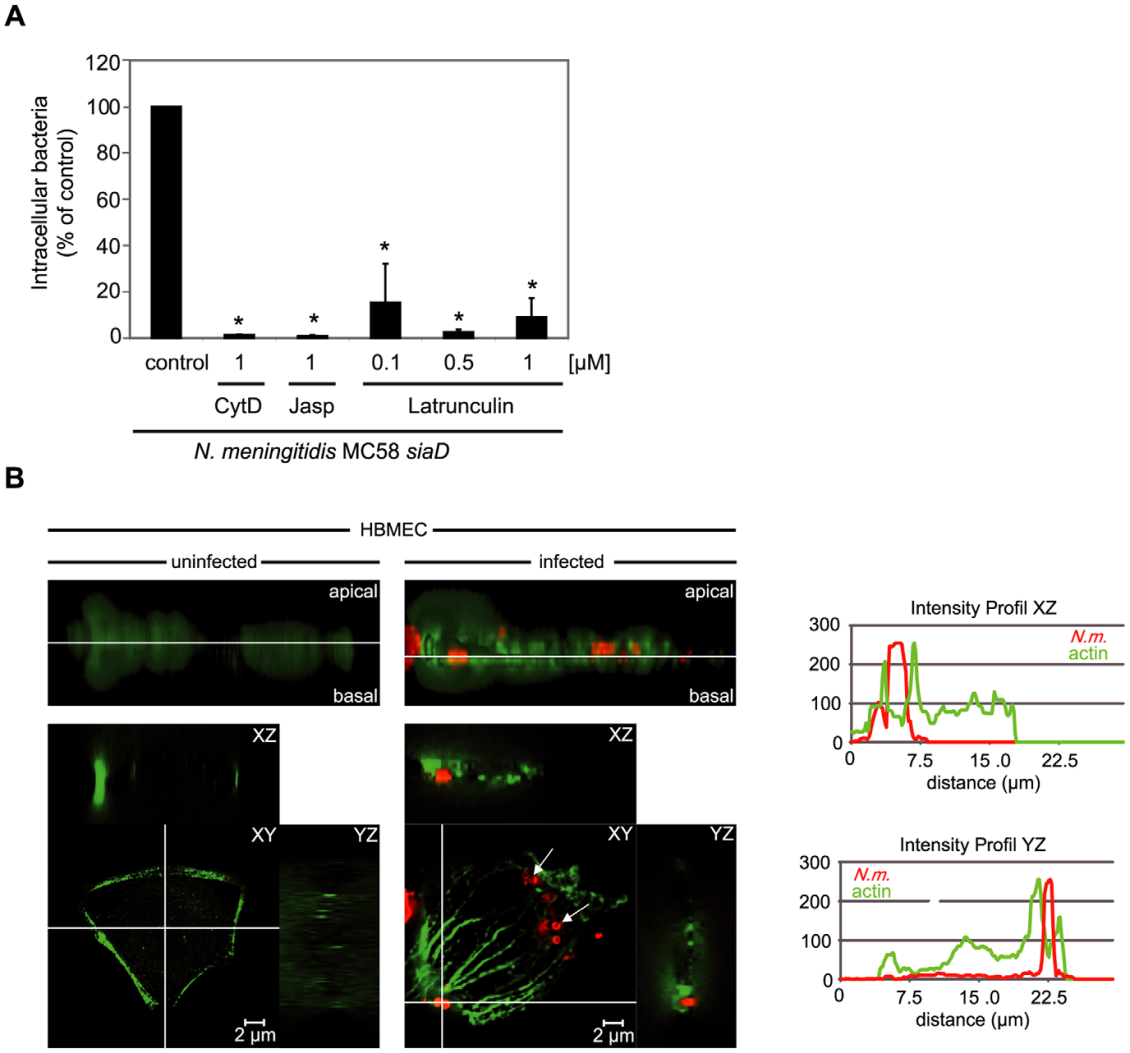
*N. meningitidis* internalization by HBMEC requires actin cytoskeleton dynamics. (A) HBMEC were pre-incubated with indicated concentrations of inhibitors of the cytoskeleton, including cytochalasin D (Cyt D, 1 µM), jaspakinolide (Jasp), Latrunculin or solvent (DMSO) as a control and infected for 8 h with the unencapsulated invasive strain *N. meningitidis* strain MC58 *siaD*. Intracellular bacteria were defined by gentamicin protection assay. The graph shows mean values +/− S.D. of three independent experiments done in duplicate. * *P*<0.05, relative to cells infected without inhibitor. (B) Immunofluorescence analysis of HBMEC infected with *N. meningitidis* for 4 h. Uninfected and infected cells were fixed and labeled with Alexa Fluor® 488 phalloidin (green fluorescence). Bacteria were immunostained with a rabbit anti-meningococcal serum and secondary TRITC-labelled goat α-rabbit IgG (red fluorescence). The upper panels represent the z-stack projections, the white lane marks the level of the xy-planes shown in the lower panels. Meningococci attached to HBMEC induced an increase of actin concentration and caused the formation of actin stress fibers, consisting of long bundles of filaments traversing the cell, whereas uninfected control cells showed no formation of stress fibers. Internalized bacteria are marked with arrows. Fluorescence signals were separately quantified in the GFP and rhodamin channels and showed an increase in the local concentration of actin adjacent to the attached bacterium. Figure shows an *X*–*Y* plan view and the *X*–*Z* and *Y*–*Z* sagittal cross-sections. Scale bar  = 2 µm.

We have previously shown that Opc/integrin-mediated internalisation of *N. meningitidis* into HBMEC depends on the rearrangement of the actin cytoskeleton, as cytochalasin D, which inhibit actin polymerization by binding with high affinity to growing ends of actin nuclei and filaments, interferes with bacterial uptake [36]. To further prove that a dynamic actin system is crucial in the internalisation process of *N. meningitidis*, we analyzed the effect of latrunculin B, which inhibits actin polymerisation by interacting with actin monomers (G-actin) and jasplakinolide, which in contrast exhibits F-actin polymerizing and stabilizing capacities. As shown in Figure 1A, both latrunculin B and jasplakinolide significantly inhibited uptake of *N. meningitidis* into HBMEC, thus demonstrating that Opc-mediated internalization requires the dynamic remodeling of actin structures.

To analyze local actin accumulation during meningococcal entry in more detail immunofluorescence analysis was performed. HBMEC were infected with *N. meningitidis* for 4 h. Infected cells were fixed and labeled with Alexa Fluor® 488 phalloidin (green fluorescence), whereas bacteria were immunostained with a rabbit α-meningococcal serum and secondary TRITC-labelled goat α-rabbit IgG (red fluorescence). As shown in figure 1B *N. meningitidis* induced the formation of actin stress fibers, consisting of long bundles of filaments traversing the cell [37], whereas uninfected control cells showed actin enrichment solely along the cell membrane. Fluorescence signals were separately quantified in the GFP (actin stain) and rhodamin channels (bacterial stain) and showed an increase in the local concentration of actin in the vicinity of the attached bacterium (Fig. 1B).

### Invasion of *N. meningitidis* into Host Cells Requires FAK Activity

We have recently demonstrated the requirement of tyrosine kinases in *N. meningitidis* uptake by HBMEC and identified c-Src PTK to be essential for this process [18]. Analyzing the tyrosine phosphorylation pattern of HBMEC lysates after infection with *N. meningitidis*, we observed an additional phosphorylated protein with an apparent molecular mass of about 125 kDa, which is within the range for the focal adhesion kinase (FAK) [18]. FAK is an important modulator of integrin-dependent focal contacts thereby orchestrating cell spreading, cell migration and integrin-initiated signaling events [28].

**Figure 2:**
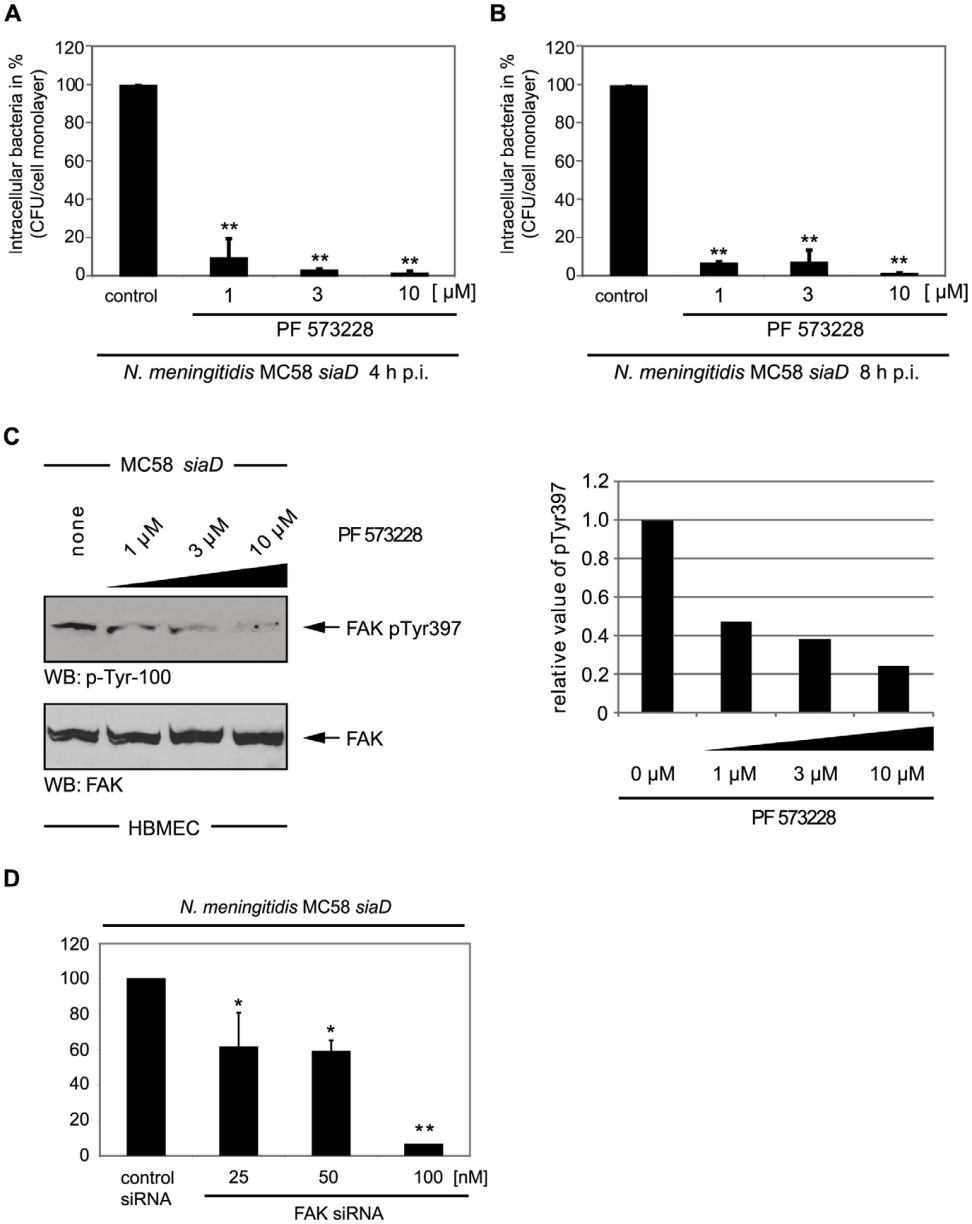
*N. meningitidis* internalization by HBMEC requires focal adhesion kinase (FAK) activity. (A and B) HBMEC were pre-incubated with indicated concentrations of the specific FAK inhibitor PF573228 and infected for 4 h and 8 h with the unencapsulated *N. meningitidis* strain MC58 *siaD*. Intracellular bacteria were defined by gentamicin protection assay. The graph shows mean values +/− S.D. of three independent experiments done in duplicate. ** *P*<0.01, relative to cells infected without inhibitor. (C) HBMEC were pre-incubated with the indicated concentrations of the FAK inhibitor PF 573228 for 1 h and infected with *N. meningitidis* MC58 *siaD* for 4 h. Cell lysates were resolved by SDS-PAGE and blotted with α-phospho-FAK Tyr^397^ demonstrating that PF 573228 blocked FAK Tyr^397^ phosphorylation in a dose-dependent manner. (D) 293T cells were transfected with indicated concentration of a commercial siRNA specific for FAK to limit FAK protein expression or transfected with unspecific control siRNA. siRNA transfected cells were infected with mutant strain MC58 *siaD* and internalized bacteria were measured by gentamicin protection assay at 4 h p.i. The graph represents mean values ± S.D. of three independent experiments done in duplicate. * *P*<0.05 and ** *P*<0.01, relative to cells transfected with the control siRNA.

To examine the involvement of FAK during the entry of *N. meningitidis* into HBMEC, invasion assays were performed in the presence and absence of the specific FAK inhibitor PF 573228 [38]. Infection assays were carried out using the invasive unencapsulated strain *N. meningitidis* MC58 *siaD* [8]. The specific FAK inhibitor PF 573228 blocked invasion by *N. meningitidis* MC58 *siaD* efficiently in a dose-dependent manner (>90% inhibition with 1 µM PF 573228 at 4 h post-infection (p.i.) and about 94% inhibition with 1 µM PF 573228 [*P*<0.01] at 8 h p.i.) (Fig. 2). To exclude the option that the observed inhibition was due to inefficient adhesion of *N. meningitidis* to HBMEC treated with PF 573228 the number of cell-associated bacteria was determined. PF 573228 treatment did not have any effect on the total number of cell-associated bacteria. Furthermore, the growth of bacteria was not affected in the presence of PF 573228 (data not shown).

We next assessed the effect of the inhibition of FAK expression using a siRNA interference approach. 293T cells were used for this genetic interference approach, because HBMEC could not be transiently transfected in a sufficient manner. *N. meningitidis* showed a similar invasion kinetic and Opc-dependent uptake mechanism in 293T compared to HBMEC, however absolute number of invasive bacteria was about one log-fold lower, probably owing to lower levels of β1 integrin expression on 293T cells compared to HBMEC as demonstrated by flow cytometry analyses in our previous study [18]. 293T cells were transfected with indicated concentration of a commercial siRNA specific for FAK to limit FAK protein expression or transfected with unspecific control siRNA. siRNA transfected cells were infected with mutant strain MC58 *siaD* and internalized bacteria were measured by gentamicin protection assay at 4 h p.i. Inhibition of FAK expression by siRNA was monitored from cell lysates from transfected cells and examined by Western blot (data not shown). As shown in figure 2D, reduced FAK expression also resulted in a strong reduction of *N. meningitidis* uptake by 293T cells (>90% inhibition with 100 nM FAK siRNA [*P*<0.01]), indicating that FAK is critical for Opc-mediated uptake of *N. meningitidis*.

### FAK is Involved in Integrin-mediated Uptake of *N. meningitidis*

**Figure 3:**
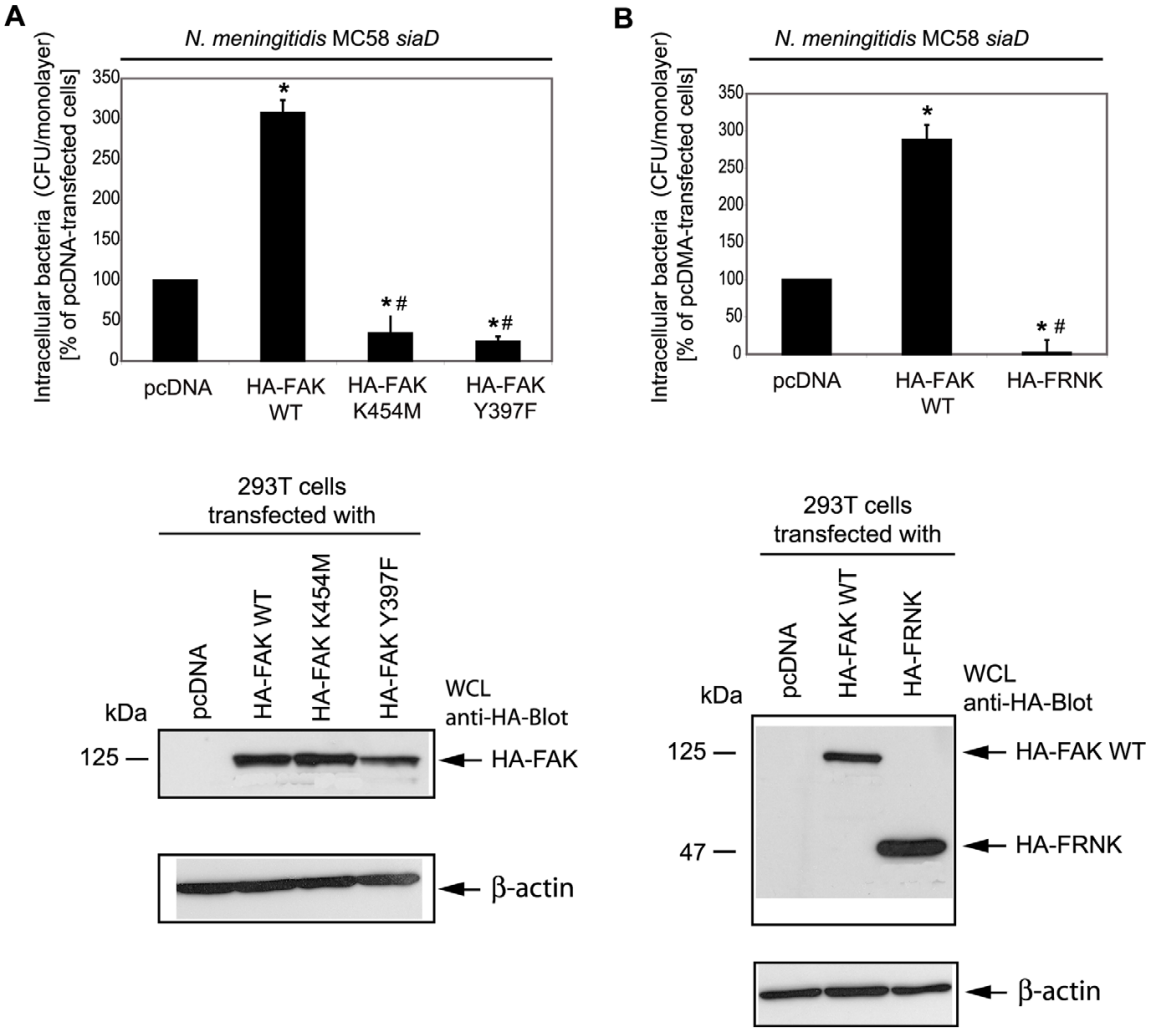
Interference with FAK functions results in decreased uptake of *N. meningitidis.* (A) 293T cells were transfected with a control plasmid (pcDNA) and a plasmid encoding wildtype FAK (HA-FAK WT) or a plasmid encoding a kinase inactive mutant (HA-FAK K454M) or a mutant that was not capable of autophosphorylation (HA-FAK Y397F). Transfected cell were infected with invasive strain MC58 *siaD* and intracellular bacteria were estimated 4 h post-infection by gentamicin protection assays. The graph represents mean values ± S.D. of three independent experiments done in duplicate. * *P*<0.05, relative to cells transfected with the control plasmid, ^#^ *P* ≤ 0.05, relative to cells transfected FAK wildtype were considered significant. In parallel, Western blotting of WCL extracts with anti-HA-tag antibody demonstrates expression of HA-tagged FAK constructs. (B) 293T cells were transfected with HA-FAK WT or a plasmid encoding the FAK related non-kinase (HA-FRNK) and infected with MC58 *siaD* as described above. The graph represents mean values ± S.D. of three independent experiments done in duplicate. * *P*<0.05. WCL were analyzed by Western blotting using an anti-HA-tag antibody and demonstrated overexpression of FRNK in transfected cells.

To analyze the role of FAK in *N. meningitidis* invasion in more detail, 293T cells were transfected with different FAK mutants that were either impaired in their kinase activity (FAK K454M) or were not capable of autophosphorylation (FAK Y397F). As a control, 293T cells were transfected with an empty control plasmid vector (pcDNA) and an expression vector encoding for wildtype FAK (FAK WT). Indeed, overexpression of cells expressing FAK K454M or FAK Y397F showed a prominent decrease in uptake of bacteria compared to wildtype FAK transfected cells (Fig. 3A).

To further confirm the findings 293T cells were transfected with mammalian expression vectors containing the FAK related non-kinase (FRNK), which has been found to be an inhibitor of FAK [39]. Overexpression of FRNK significantly (>95%, *P*<0.05) inhibited *N. meningitidis* invasion into 293T compared to 293T cells transfected with the empty control vector as well as compared to 293T cells transfected with FAK WT (*P*<0.01) (Fig. 3B), together indicating that FAK is involved in integrin-mediated uptake of *N. meningitidis*.

### FAK-deficient Fibroblasts are Significantly Less Invaded by *N. meningitidis*

**Figure 4:**
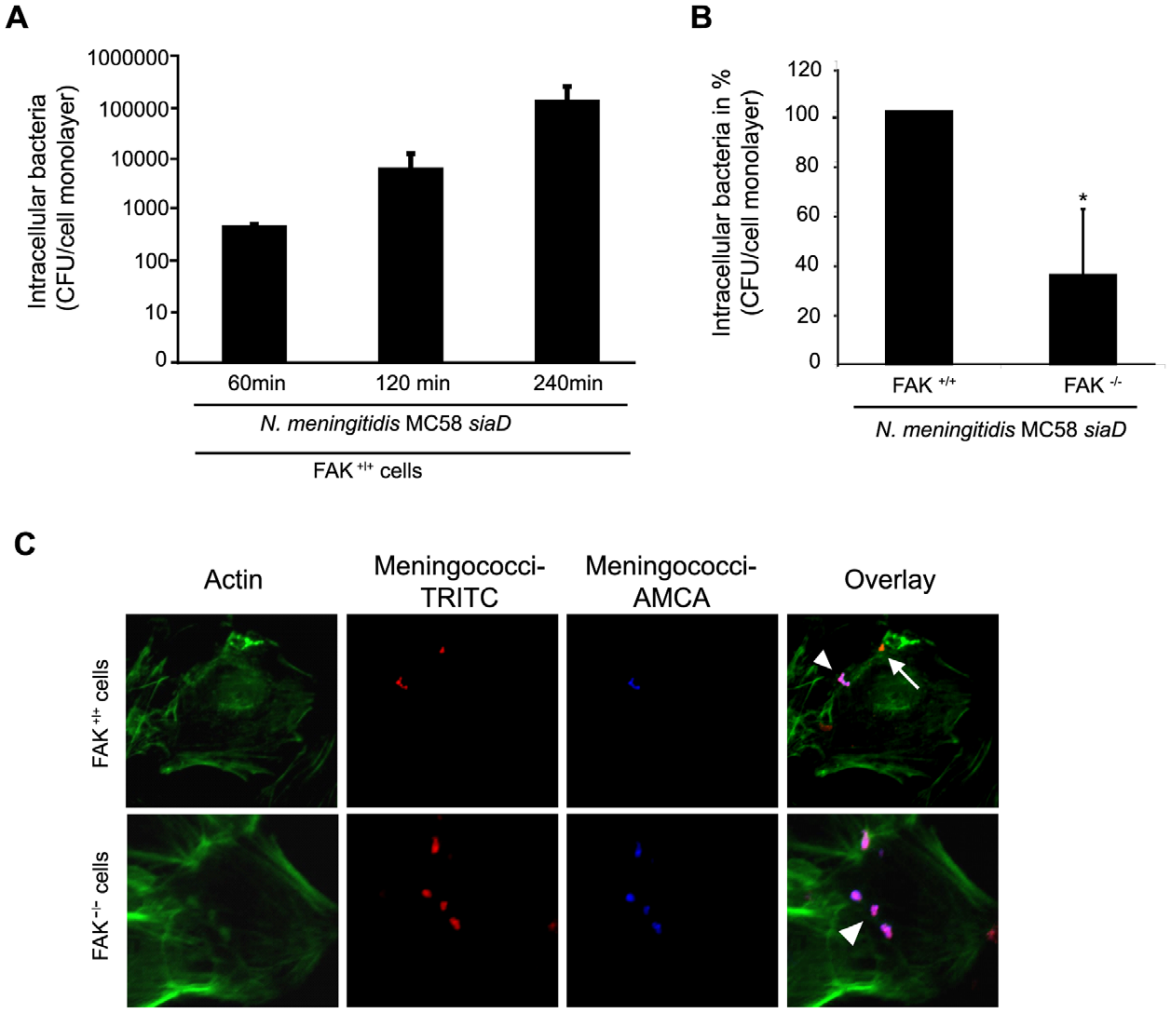
FAK-deficient cells are impaired in their ability to internalize *N. meningitidis.* (A) FAK^+/+^ fibroblasts were infected with MC58 *siaD* at an MOI of 30 in presence of RPMI cell culture medium, supplemented with 10% human serum (HS). Intracellular bacteria were defined after gentamicin treatment at 60, 120 and 240 min post-infection (p.i.) demonstrating an invasion kinetic similar to 293T cells and HBMEC. The graphs represent mean value ± S.D. of three different independent experiments done in duplicate. * *P*<0.05. (B) FAK re-expressing (FAk^+/+^) and FAK-deficient (FAK^−/−^) fibroblasts were infected with invasive strain MC58 *siaD* in HS-supplemented RPMI cell culture medium. Intracellular bacteria were estimated at 4 h p.i. by gentamicin protection assays. The graph represents mean value ± S.D. of three different independent experiments done in duplicate. * *P*<0.05. (C) FAK^+/+^ and FAK^−/−^ fibroblasts were infected with *N. meningitidis* strain MC58 *siaD* for 4 h and analyzed by immunofluorescence microscopy. Extracellular bacteria (arrowhead) stain positive with both TRITC (red fluorescence) and AMCA (blue fluorescence), whereas intracellular bacteria (arrow) are labeled with TRITC only. Cell actin was stained with Alexa Fluor® 488 phalloidin (green fluorescence).

The previous results suggested an important role of FAK by activating cytoskeleton remodelling in *N. meningitidis* invasion. We therefore postulated that cells lacking FAK should not support *N. meningitidis* invasion. Mouse fibroblasts derived from FAK-deficient embryos (FAK^−/−^ cells) and HA-FAK re-expressing cells (FAK^+/+^ cells) were employed in gentamicin protection assays. Importantly, in FAK re-expressing cells the kinetics of invasion of *N. meningitidis* was comparable to that of HBMEC (Fig. 4A) [8].

FAK-deficient fibroblasts were infected with *N. meningitidis* MC58 *siaD* and resulted in significant decrease of invasive bacteria (Fig. 4B), verifying the critical role of FAK in *N. meningitidis* invasion. To further prove data observed in the protection assay, differential fluorescence staining of intracellular and extracellular bacteria in infected FAK^+/+^ and FAK^−/−^ cultures was performed. Immunfluorescence microscopy revealed that after 4 h p.i. a large portion of cell-associated meningococci was localised inside the FAK^+/+^ cells, whereas less intracellular bacteria were found in FAK^−/−^ cells (Fig. 4C). When we calculated the percent of intracellular stained bacteria per 20 HBMEC cells immunofluorescence analyses revealed a similar proportion of invasive bacteria as shown by gentamicin protection assays (data not shown). Furthermore, infected cultures of both FAK^+/+^ and FAK^−/−^ cells showed equivalent amounts of bacteria that attached to the surface of the cells.

### *N. meningitidis* Induces Enhanced Tyrosine Phosphorylation of Cellular Proteins in FAK^+/+^ Fibroblasts

Recruitment of FAK to focal contacts is associated with increased FAK tyrosine phosphorylation, which in turn results in enhanced tyrosine phosphorylation of downstream effectors. To further gain insight into the tyrosine phosphorylation pattern of cellular proteins in response to *N. meningitidis* infection involving FAK activity, FAK^+/+^ and FAK-deficient FAK^−/−^ fibroblasts were infected with *N. meningitidis* MC58 *siaD.* FAK^+/+^ and FAK^−/−^ cells were serum starved and plated on poly-L-lysine coated dishes to minimize integrin engagement by the cell culture substrate. FAK^+/+^ and FAK^−/−^ cells were left uninfected or infected with *N. meningitidis* MC58 *siaD* for a 8 h period and cell lysates were collected at 30 min, 60 min, 120 min, 240 min and 480 min p.i. and analyzed by Western blotting using the α-p-Tyr-100 antibody.

**Figure 5:**
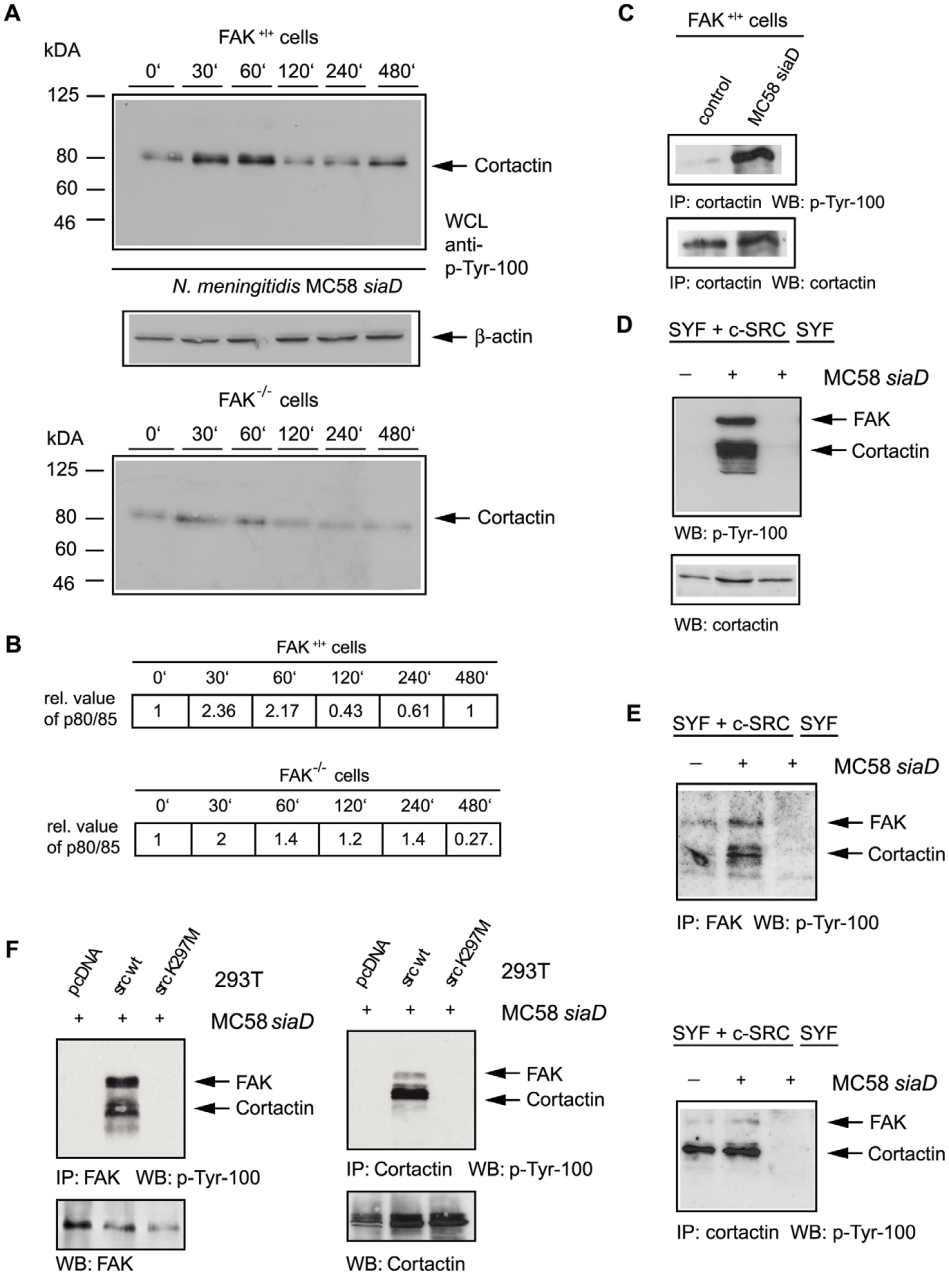
*N. meningitidis* induced increased tyrosine phosphorylation of cellular proteins. (A) FAK^+/+^ cells were serum starved and plated on poly-L-lysine coated dishes. Fibroblasts were left uninfected or infected with *N. meningitidis* MC58 *siaD* for a 8 h period and cell lysated were collected at from uninfected control cells and at 30 min, 60 min, 120 min, 240 min and 480 min post-infection and analyzed by Western blotting using the anti-phosphotyrosine antibody p-Tyr-100. (B) The increase of the phosphorylated protein was quantified by densitometric analysis and increase was estimated by comparison to the uninfected control. Densitometric analysis was performed as described in Experimental procedures. Staining of the samples with anti-actin antibody was used as loading control. (C) FAK^+/+^ cells were infected for 4 h as described above and samples were immunoprecipitated (IP) with an anti-cortactin antibody and immunoprecipitates were analyzed with α-p-Tyr-100 antibody (upper panel). Membranes were stripped and reprobed with polyclonal anti-cortactin antibody (lower panel). (D) Src re-expressing (SYF + c-Src) and c-Src-deficient (SYF) fibroblasts were infected with invasive strain MC58 *siaD* for 4 h as described above. Cell lysates were collected and analyzed by Western blotting using antibody p-Tyr-100. After stripping membranes were re-probed with polyclonal anti-cortactin antibody (lower panel). (E) SYF + c-Src and SYF cells were infected with strain MC58 *siaD* for 4 h and samples were immunoprecipitated either with an α-FAK antibody (Fig. 5E, upper panel) or α-cortactin antibody (Fig. 5E, lower panel) and immunoprecipitates were analyzed with α-p-tyr-100 antibody. (F) 293T cells were transfected with the empty control vector (pcDNA), a plasmid encoding wild-type c-Src and a vector encoding the inactive version of Src [Src K297M] to prove that Src kinase activity is required FAK/cortactin phosphorylation. Transfected cells were infected as described above, followed by an IP with an α-FAK antibody or α-cortactin antibody, respectively. Western blot analysis with α-p-tyr-100 demonstrated that Src kinase activity is required FAK/cortactin phosphorylation.

As shown in figure 5, meningococci induced tyrosine phosphorylation of a protein with an apparent molecular mass of 80 kDa. Tyrosine phosphorylation of the 80-kDa band increased in a time dependent manner upon infection with *N. meningitidis* (Fig. 5A). Densitometric analyses revealed that phosphorylation of the 80-kDa protein peaked at 120 min p.i. (Fig. 5B), corresponding to time point of bacterial internalisation. Densitometric analyses also revealed a small increase in tyrosine phosphorylation detectable in FAK^−/−^ cells that might be due to Src-mediated cortactin phosphorylation.

Since the size of the 80-kDa protein present in FAK^+/+^ cells is in range of the size of the FAK/Src complex substrate cortactin (80/85-kDa), we next determined, whether cortactin is phosphorylated in response to integrin-mediated uptake of *N. meningitidis* invasion process. Therefore, FAK^+/+^ cells were infected as described above and samples were immunoprecipitated with α-cortactin antibody and precipitates were analyzed with α-p-Tyr-100 antibody. As shown in figure 5C tyrosine phosphorylation of cortactin was increased following stimulation with *N. meningitidis*. Membranes were stripped and re-probed with α-cortactin antibody to confirm the presence of equal amounts of cortactin protein (Fig. 5C).

Cortactin is a major substrate of Src kinase. And we have recently shown that *N. meningitidis* infection activates Src in host cells [18]. We next proved whether Src kinase activity in response to *N. meningitidis* alone is sufficient to phosphorylate cortactin and whether FAK activity can compensate for Src. We therefore applied fibroblasts (SYF cells) which were derived from Src, Yes and Fyn-deficient mouse embryos [40]. SYF cells re-expressing c-Src (SYF + c-Src cells) were used as control. Both Src PTK-deficient SYF cells and SYF cells re-expressing c-Src were infected as described above and analyzed by Western blotting using the α-p-Tyr-100 antibody. As shown in figures 5D and 5E, tyrosine phosphorylation of cortactin was increased in infected SYF cells re-expressing c-Src, whereas there was no increase in phosphorylation of cortactin and FAK detectable in infected SYF cells. To further prove that Src kinase activity is required for cortactin/FAK phosphorylation we included a kinase-inactive version of Src [Src K297M] in the following experiments as described in our previous study [18]. 293T cells were either transfected with an empty control vector (pcDNA), a plasmid encoding wild-type c-Src and a vector encoding the inactive version of Src [Src K297M]. Transfected cells were infected as described above, followed by an IP with an α-FAK antibody or α-cortactin antibody, respectively. Western blot analysis with α-p-tyr-100 demonstrated that Src kinase activity is required for cortactin/FAK phosphorylation in response to *N. meningitidis* infection. Together theses results indicate that Src activity alone is sufficient to activate cortactin, and FAK cannot compensate for the loss of Src activity.

### Interference with Cortactin Function Significantly Reduces Cellular Invasion by *N. meningitidis*

**Figure 6:**
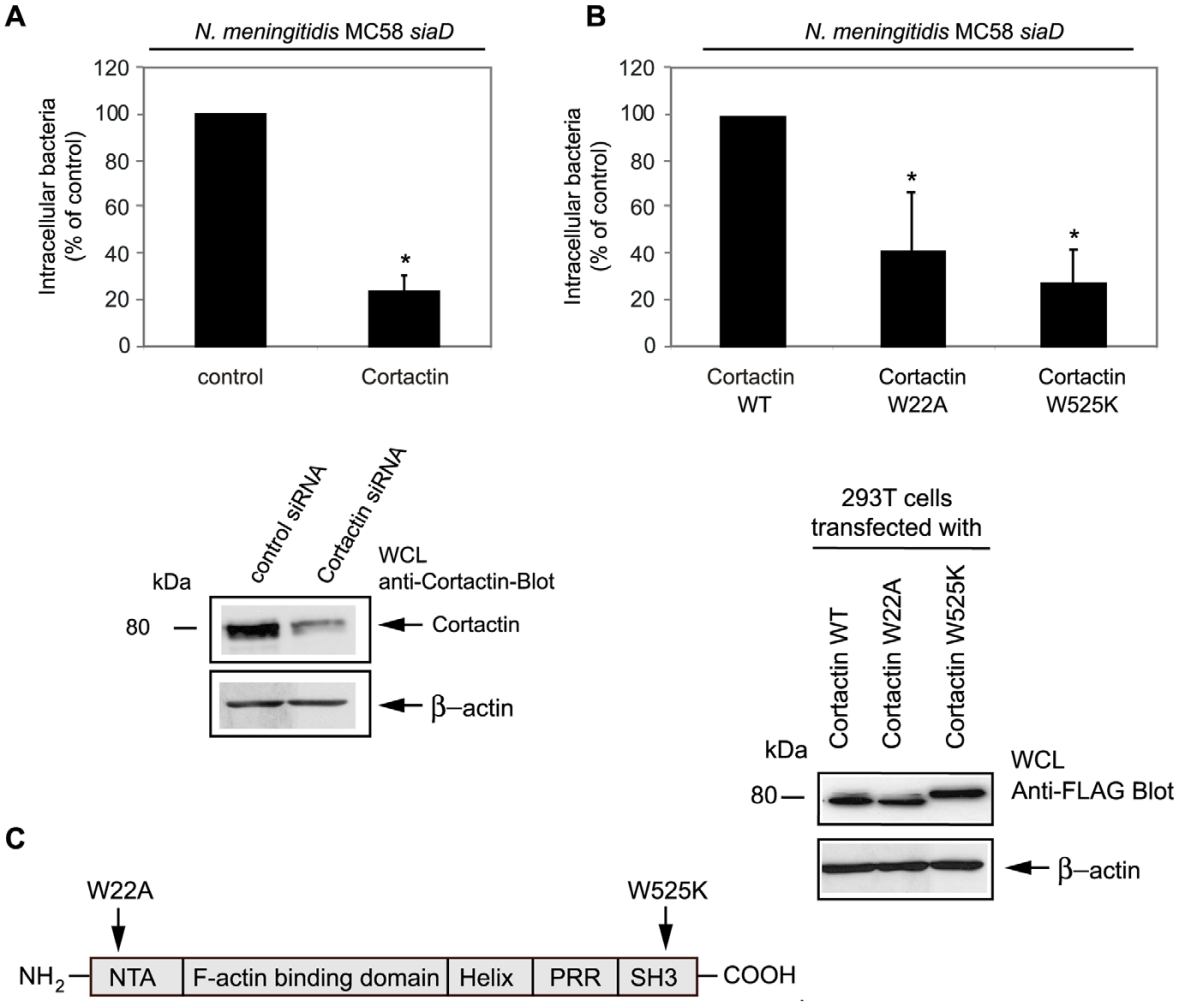
Interferences with cortactin expression results in diminished uptake of *N. meningitidis* into host cells. (A) 293T cells were transfected with equal amounts (25 nM) of cortactin and control-siRNA. Invasion assays were performed 72 h post transfection with invasive strain MC58 *siaD* and intracellular bacteria were estimated 8 h post-infection by gentamicin protection assays. The graph represents mean values ± S.D. of three independent experiments done in duplicate. * *P*<0.05, relative to cells transfected with the control siRNA. In parallel whole cell lysates (WCL) extracts were prepared and subjected to Western blot analysis. Staining with polyclonal anti-cortactin antibody demonstrated genetic knock-down expression of cortactin in cortactin-siRNA-transfected cells compared to control siRNA-transfected cells. (B) 293T cells were transiently transfected with wildtype cortactin (cortactin WT), a plasmid encoding a mutant form of cortactin interfering with Arp2/3 association (W22A) or a plasmid encoding a mutant form of cortactin interfering with dynamin binding (W525K). Transfected cells were infected with strain MC58 *siaD* and the numbers of intracellular bacteria were determined after gentamicin treatment. The data in the graph are the mean values ± S.D. of three independent experiments done in duplicate. * *P*<0.05, relative to cells transfected with cortactin WT. Western blotting of WCL with anti-FLAG antibody demonstrated that there was equal overexpression of the wildtype cortactin and both cortactin mutant forms. The FLAG-tagged wildtype cortactin runs higher than endogenous cortactin, and the W525K mutant is anomalously high in SDS gels, as described elsewhere [72]. (C) Schematic representation of the domain structure of cortactin. The cortactin point mutations are indicated. W22A, tryptophan to alanine point mutation in the NTA domain; and W525K, tryptophan to lysine point mutation in the SH3 domain. Domains include: NTA, N-terminal acidic (NTA) region; PRR, proline-rich region; and the SH3 domain that comprises the carboxy terminus.

Cortactin has emerged as a key component involved in the coordination of membrane dynamics and cytoskeleton remodelling [41]. We next focused on the role of cortactin, which is a major subject to tyrosine phosphorylation of the FAK/Src complex. Although a role of cortactin in the uptake process of *N. meningitidis* has been described before [42], a role of the functional domains that mediate Arp2/3 or dynamin binding in the internalization process has not been elucidated. We first repeated the finding that cortactin has a functional role in our model system using a siRNA interference approach. As shown for role of FAK we used 293T cells for the following genetic interference approaches, because 293T cells can easily be transfected. 293T cells were transfected with a commercial siRNA that corresponds to the human cortactin gene to limit cortactin protein expression. siRNA transfected cells were infected with mutant strain MC58 *siaD* and internalized bacteria were measured by gentamicin protection assay. Inhibition of cortactin expression by siRNA was monitored from cell lysates from transfected cells and examined by Western blot. As shown in figure 6, cortactin protein was significantly less expressed in cells transfected with cortactin siRNA compared to control-transfected cells (Fig. 6A). The reduced cortactin expression also resulted in a strong reduction of *N. meningitidis* uptake by 293T cells (about 80%), verifying the functional role for this actin binding protein (Fig. 6A).

Cortactin binds to and acts as an activator of the Arp2/3 complex, which mediates *de novo* F-actin nucleation and F-actin branch assembly [35], [43]–[45]. The C-terminus of cortactin contains a Src homology 3 (SH3) domain, that interacts with prolin-rich binding sequences in several cortactin interacting partners, including WIP, N-WASP, MLCK and dynamin-2. To examine the functional importance of these two domains of cortactin in meningococcal uptake, we employed mammalian expression vectors either containing a mutant form of cortactin in the NTA domain, which is no longer able to bind the Arp2/3 complex (cortactin W22A, tryptophan to alanine point mutation) and a mutant form impaired in the ability to bind dynamin-2 (cortactin W525K, tryptophan to lysine point mutation in the SH3 domain) (Fig. 6C). 293T cells were transiently transfected either with cortactin wildtype or with both cortactin mutant forms, which were both well expressed as demonstrated by Western blotting (Fig. 6B). Interestingly, overexpression of both mutants resulted in a prominent decrease in uptake of *N. meningitidis* (Fig. 6B). To further address that the mammalian expression vectors explored in this study did not bind their binding partners Arp2/3 or dynamin, 293T cells were again transiently transfected with the mutant form cortactin W22A or cortactin W525K, respectively, followed by an α-cortactin IP. Immunoprecipitates were analyzed with an α-Arp2/3 or α-dynamin antibody and revealed that dynamin indeed did not bind to the cortactin W525K construct any more (Fig. S1). The Arp2/3 complex however still bound to the cortactin W22A construct, however binding was significantly less compared to cortactin W525K construct (Fig. S1). Together these results suggest that both domains are critical for efficient uptake of *N. meningitidis* into eukaryotic cells.

### The Entry Site of *N. meningitidis* is Enriched in Cortactin

**Figure 7:**
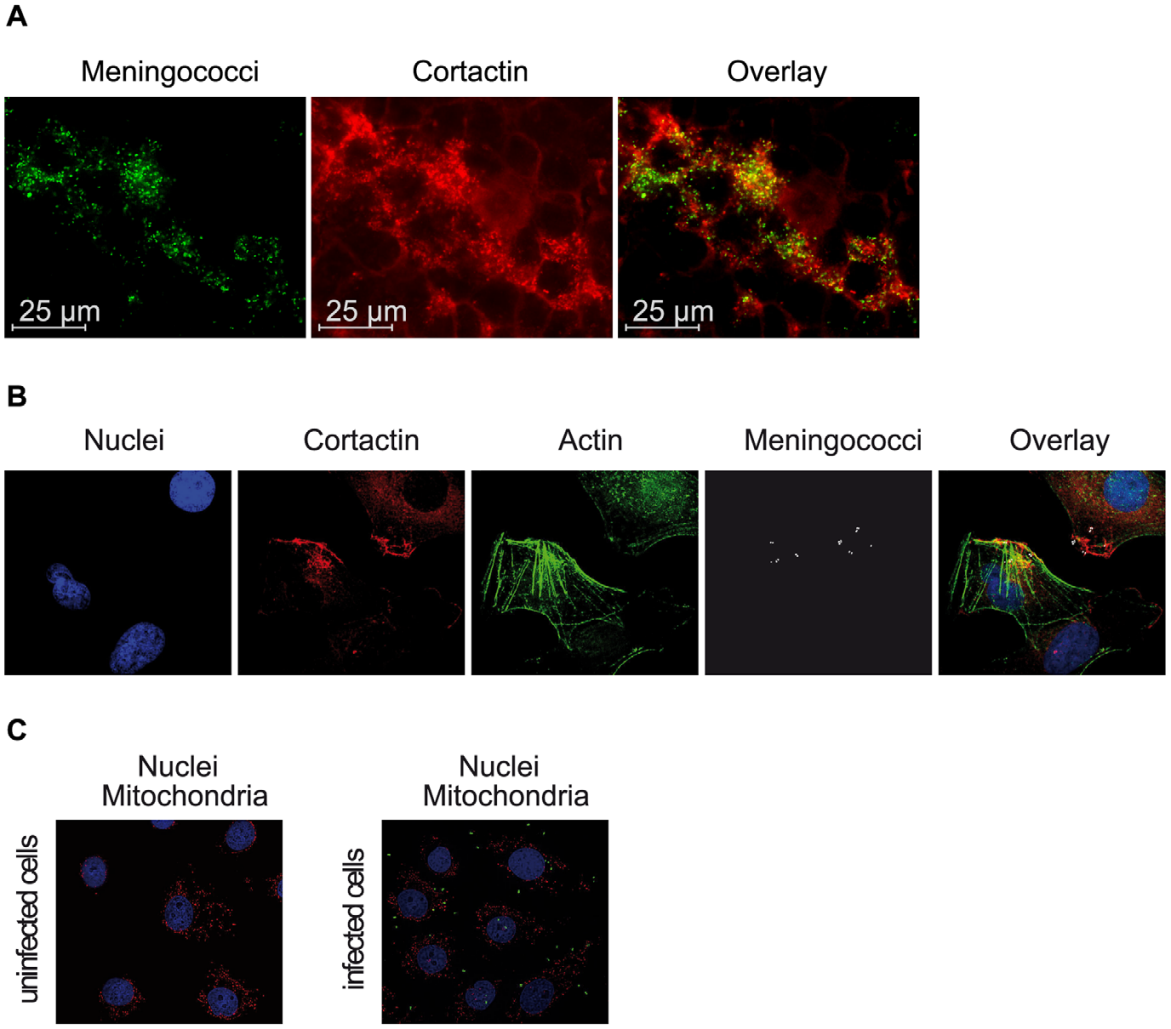
*N. meningitidis* induces recruitment of cortactin. (A) 293T cells were infected for 4 h with FITC-labeled meningococci (green fluorescence). Infected cells were fixed and stained with an anti-cortactin antibody followed by secondary α-rabbit TRITC-conjugated antibody (red fluorescence). Immunofluorescence demonstrated local recruitment of cortactin to cell-associated bacteria. Scale bars represent 25 µm. (B) For the close-up view, cells were infected with *N. meningitidis* for 4 h, fixed and incubated with DAPI (blue fluorescence), Alexa Fluor® 488 phalloidin (green fluorescence), cortactin goat α-rabbit/TRITC-conjugated goat α-rabbit (red fluorescence) and α-meningococcal serum/Alexa Fluor® 594 goat α-mouse (pseudocolored in gray). The immunofluorescence confirmed an accumulation of cortactin and the formation of stress fibers adjacent of bacterial attachment. (C) To exclude that bacterial attachment occurred solely on dead cells, cells were either infected with FITC-labeled bacteria or left uninfected and were incubated with MitoTracker (red fluorescence) and DAPI. No signs of apoptosis could be observed before or after 4 h of infection.

To finally investigate if cortactin is one of the tyrosine kinase substrate recruited to the site of meningococcal entry, 293T cells were infected with FITC-labeled invasive strain *N. meningitidis* MC58 *siaD* for 4 h. As shown in figure 7 A and B, *N. meningitidis* induced a significant recruitment of cortactin to the site of bacterial attachment. To exclude that bacterial attachment occurred solely on dead cells, cells were either infected with FITC-labeled bacteria or left uninfected and were incubated with MitoTracker and DAPI. MitoTracker accumulates in active mitochondria and diffuses by disturbances of mitochondrial respiration chain back in the cytoplasm and is therefore a very sensitive marker for cell damage [46]. As shown in figure 7C, no signs of apoptosis could be observed in infected cells.

## Discussion

Our previous results demonstrated that an intact actin cytoskeleton is required for efficient *N. meningitidis* internalization, as cytochalasin D, an inhibitor of actin polymerization, significantly blocked invasion of *N. meningitidis* into human brain endothelial cells (HBMEC) [18], [36]. Here we show, that both latrunculin B and jasplakinolide significantly inhibited uptake of *N. meningitidis* into HBMEC. Both inhibiting actin monomer incorporation into F-actin by latrunculin B as well as inhibiting the depolymerization of F-actin by jasplakinolide abolish the dynamic turnover of actin cytoskeleton structures. Therefore, Opc-mediated internalization requires the dynamic remodeling of actin structures.

In this study, we furthermore demonstrate that *N. meningitidis* entry induces actin condensation at the vicinity of the bacterium suggesting that invasive *N. meningitidis* transduces signals for cytoskeleton reorganisation and for the formation of focal contacts. In addition, previous data for assays using genistein revealed that protein tyrosine kinases (PTKs) play a significant role in *N. meningitidis* uptake by HBMEC. We identified c-Src as an important signal protein in this process, which is activated as a downstream result of integrin engagement by the meningococcus [18]. By analyzing the tyrosine phosphorylation pattern of HBMEC cell lysates after infection with *N. meningitidis*, we observed enhanced phosphorylation of a protein with an apparent molecular mass of about 125 kDa, which is within the range for the focal adhesion kinase (FAK). Based on these results, we investigated the role of FAK for *N. meningitidis* invasion initiated by Opc-mediated binding to the integrins in detail.

Our initial data obtained using the specific FAK inhibitor PF 573882 indicated that FAK has a significant role in invasion of HBMEC by *N. meningitidis*. The observed effect of PF 573882 on *N. meningitidis* invasion was not the result of diminished adhesion to HBMEC since PF 573882-treated cells did not differ in the number of adherent bacteria compared to untreated cells.

FAK is an important modulator of integrin-dependent focal contacts thereby orchestrating cell spreading, cell migration and integrin-initiated signaling events [28]. Phosphorylation of different tyrosine residues of FAK increases its ability to interact with a number of signaling molecules via its Src Homology (SH) 2 binding domain, such as the Src kinase family members, phosphatidylinositol 3-kinase (PI-3 kinase) and Grb2. The autophosphorylation site of FAK (Tyr397) is required for the association with the SH2 binding domain of Src familiy kinases and of the p85 subunit of PI-3 kinase. Here we showed that overexpression of a mammalian expression vector encoding an autophosphorylation mutant of FAK (FAK Y397F) diminished bacterial uptake into eukaryotic cells, demonstrating that autophosphorylation is important for the invasion process. Further genetic interference with this non-receptor kinase using a kinase inactive version of FAK (FAK K454M) also decreased bacterial uptake, indicating that FAK kinase activity is critical. Importantly, FAK-deficient fibroblasts were significantly less invaded by meningococci. In addition, overexpression of FRNK, a negative regulator of FAK, inhibited *N. meningitidis* invasion into eukaryotic cells. FRNK is strongly recruited to focal contacts and thereby displaces FAK from the binding partners of its focal adhesion binding (FAT) domain, which results in dissociation of integrin control and loss of activation.

Since our previous study revealed that Src kinase activity is essential for *N. meningitidis* invasion, the data shown here provide evidence that the catalytic activity of both FAK and the Src kinase is required in this process. Involvement of FAK in bacterial uptake has been observed for other pathogens [47]–[50]. FAK can be activated downstream of integrin engagement or other receptors [47]. For example, FAK and Src have been demonstrated to be involved in invasin/integrin-mediated uptake of *Yersinia* spp. [51]–[53]. Similar to *N. meningitidis, S. aureus* recruits fibronectin via fibronectin binding proteins (FnBP) to its surface, which act as a molecular bridge linking staphylococci to integrins. FnBP-mediated engagement of integrins initiates clustering of integrins and the internalisation of the bacteria into host cells involving both FAK and Src kinase activity [49], [54].

Invasion into brain endothelial cells has been analyzed for the meningitis-causing pathogen *E. coli* in detail [55]. In contrast to *N. meningitidis*, *E. coli* uptake into HBMEC is integrin-independent, and though invasion requires FAK activity, Src kinase activity is not necessary [47], suggesting that these two meningitis causing pathogens have evolved different strategies to invade brain endothelial cells and to overcome the blood-brain barrier.

FAK and c-Src have been demonstrated to form a dual kinase complex, which binds to and can phosphorylate various adaptor proteins such as p130Cas and paxillin. So far, at least 12 different adaptor proteins have been described [56]. Among these molecules, tensin, filamin, talin, plectin and α-actinin can provide a direct link to the actin cytoskeleton. Analysing the phosphorylation pattern in FAK^+/+^ expressing cells, we demonstrated that infection with *N. meningitidis* also induced enhanced tyrosine phosphorylation of the actin-filament binding protein cortactin. Cortactin is an actin filament-binding protein and a major substrate of Src kinase. Dynamic changes of the cortical actin cytoskeleton are pivotal for several cellular events, including cell motility, endocytosis and phagocytosis and movement of intracellular particles [45] and cortactin is a central regulator of the host cytoskeleton. In the context of bacterial infection several studies revealed that reorganisation of cortical actin cytoskeleton is required for bacterial adhesion and uptake. Hence it was called an Achilles’ heel of the actin cytoskeleton [57].

Recruitment of cortactin to the site of *N. meningitidis* entry has nicely been demonstrated by Hoffmann *et al.* [58]. They showed that cortactin is phosphorylated upon infection with meningococci and that phosphorylation is crucial for efficient invasion. Consistent with this study, we observed that cortactin is recruited to the entry site of meningococci that involves an integrin-mediated uptake mechanism. Cortactin localizes to the cellular protrusions, which surround *N. meningitidis* microcolonies and form the entry site responsible for bacterial uptake. In this study, we furthermore aimed to examine the contribution of specific domains of cortactin to meningococcal invasion. Our study suggested that two domains of cortactin, comprising the NTA domain and the SH3 domain are critical in this process. Employing a cortactin mutant defective for binding to the Arp2/3 complex, we could show that overexpression of this mutant significantly reduced uptake of bacteria suggesting that this domain is involved in actin cytoskeleton reorganization probably by directly involving the activation of the Arp2/3 actin polymerization machinery.

Cortactin is also a key component of the endocytotic macherinery as well. It participates in the invagination of the forming endosomes and vesicle scission though interaction with dynamin, a large GTPase implicated in the budding and scission nascent vesicles from parent membranes [59]. By using a cortactin mutant defective for dynamin binding our data are indicative that a cortactin-dynamin complex has a substantial impact on the entry process. The amino exchange W525K occurs in the SH3 domain of cortactin that interacts with proline-rich binding sequences in several cortactin interaction partners, including WIP, N-WASP, MLCK and dynamin-2. We cannot exclude that proper binding to other interactions partners is influenced. Thus, the precise role of the Arp2/3 complex and dynamin in the entry process of *N. meningitidis* has to be elucidated in future experiments.

Interestingly, a study by Backert and co-workers recently discovered FAK as a binding partner of cortactin based on immunofluorescence and immunoprecipitation experiments using transfection and infection with *Helicobacter pylori* as a model organism. They demonstrated that FAK is in complex with cortactin [60]. The interaction occurs within the SH3 domain of cortactin and involves a so called PR3 (proline-rich 3) region of FAK, resulting in activation of FAK. In the light of these findings, the significant decrease of bacterial uptake in cortactin W525K overexpressing cells might be also explained by an interference with the interaction of the SH3 domain with FAK resulting in decreased activation of FAK and thus bacterial invasion observed in our experiments.

Cortactin contributes to two mechanisms in the interaction of specific pathogens with host cells. First, cortactin is implicated in the reorganisation of cortical actin cytoskeleton and second, cortactin is involved in formation of actin tails which are used by pathogens (e.g. *Listeria*, *Shigella*) for movement within and between the cells. For example, cortactin is recruited to the pedestral formation during adhesion of enteropathogenic and enterohaemorrhagic *Escherichia coli* (EPEC and EHEC) [61], [62], it localizes to the cellular protrusions which from the entry structure for *Shigella flexneri* uptake [63] and to the interaction site between *Cryptosporidium parvum* and the host cell [64]. Cortactin tyrosine phosphorylation is dependent on the pathogen: it occurs upon infection with *Shigella*, Vaccinia virus and *Cryptosporidium* [63]–[65], whereas cortactin phosphorylation is not necessary for uptake of EPEC and EHEC [61]. The gastric pathogen *H. pylori* in contrast induces the suppression of tyrosine-phosphorylated cortactin [60].

Actin cytoskeletal structures consist of cortical actin, stress fibers, lamellipoda, filopodia and microspikes. The reorganisation of preexisting actin filaments into these structures is mediated especially by members of the Rho family GTPases and RhoA activation leads to the formation of actin stress fibers, which are linked to the integrin at the inner face of the plasma membrane at the focal adhesion. Focal adhesions consist of about 165 proteins acting in concert as a mechanosensor to transduce and transmit mechanical information between the cell exterior and cell interior in a bidirectional way [56]. Focal adhesion composition is dynamic with numerous components incooperated and removed over time. The main components are integrins, vinculin, talin, paxillin, α-actinin, FAK, zyxin and tensin. FAK, zyxin and tensin are exclusively found in focal adhesion and not in focal complexes [66], which evolve from nascent adhesion sites or focal points and mature into focal adhesions. Future studies will be directed towards elucidating the precise role of these focal contact proteins and their interplay in the interaction between *N. meningitidis* and the host cell and how they function in actin cytoskeleton rearrangement necessary for bacterial internalization.

In summary, we have demonstrated that FAK activity is required for meningococcal invasion and the autophosphorylation and kinase activity of FAK are essential for the uptake of *N. meningitidis* by the host cell. In addition, meningococcal infection leads to cortactin phosphorylation. While Src activity alone is sufficient to activate cortactin, FAK cannot compensate for the loss of Src activity. Mutation of critical cortactin amino acids either within the domain that interacts with dynamin or within the NTA domain that activates the Arp2/3 complex support the hypothesis that both domains are critical for efficient bacterial uptake. Together, these data provide evidence that Src, cortactin and FAK are required for Opc-mediated cell invasion by *N. meningitidis*.

## Materials and Methods

### Bacterial Strains and Growth Conditions

#### Bacterial strains

*Neisseria meningitidis* strain MC58 (B15:P1.7,16b) [67], and isogenic unencapsulated mutant strains MC58 *siaD* and MC58 *siaD*, *opc* [8] were routinely cultured in proteose-peptone medium supplemented with 1% Polyvitex (bioMerieux, Lyon, France) to the mid-logarithmic phase and diluted to approximately 1×10^7^ colony-forming units (CFU) in RPMI 1640 medium (Biochrom AG, Berlin, Germany) supplemented with 10% heat-inactivated (30 min at 56°C) human serum (HS) for all cell culture experiments.

### Cell Culture, Infection Assay

Human brain microvascular endothelial cells (HBMEC) were kindly provided by K. S. Kim (Baltimore, USA) [68] and cultured as described recently [8]. Human embryo kidney cell line 239T was cultured in DMEM (Biochrom, Berlin, Germany) with 10% fetal calf serum (FCS) at 37°C, 5% CO_2_. Fibroblasts derived from FAK knockout mouse embryos (FAK^−/−^) and FAK re-expressing cells (FAK^+/+^; DA2 cells) [69] were cultured in DMEM/10% FCS supplemented with 1% non-essential amino acids on gelatin-coated (0.1% in PBS) cell culture dishes/flasks. Fibroblast derived from with Src, Yes, Fyn triple knock out mouse embryos (SYF cells, [40]) were kindly provided by P. Soriano (FHCRC, Seattle, WA). SYF cells re-expressing c-Src (SYF + c-Src cells) were used as a control. All cell cultures were incubated at 37°C with 5% CO_2_.

### Inhibitors and Antibodies

The actin filament function inhibitors cytochalasin D, jaspakinolide and latrunculin B were purchased from Calbiochem (La Jolla, CA, USA). FAK inhibitor PF 573228 was from Tocris (Tocris Bioscience, Bristol, UK). Inhibitors were reconstituted as recommended. Inhibitors were added 30 min prior to infection and were present during the infection process.

For Western blot and immunofluorescence analyses the following antibodies were used: phosphotyrosine mouse monoclonal antibody (mAb) p-Tyr-100 (9411) (Cell Signaling Technology, Danvers, MA), mAb against HA-tag (clone 12CA5) (abcam, Cambridge, UK), mAb against FLAG-tag (clone M2) (Sigma-Aldrich (Sigma, St Louis, Mo), rabbit polyclonal cortactin antibody (H-191) (Santa Cruz Biotechnology, Santa Cruz, CA) and α-phospho-FAK Tyr^397^ (clone 2D11) (nanoTools, Teningen, Germany). For immunofluorescence analyses Alexa Fluor® 488 phalloidin (Molecular Probes/Invitrogen), rabbit α-meningococcal serum [8] and secondary rhodamine (TRITC)-conjugated antibody (Dianova) were used.

### Infection Experiments and Gentamicin Protection Assay

For invasion assays, HBMEC, 293T cells or fibroblasts were seeded onto 24-well tissue culture plates (Corning Costar) at a density of 5×10^4^ cells per well and were grown to ≈1×10^5^ prior to infection. Cells were infected with bacteria at a multiplicity of infection (MOI) of 30 either in presence of RPMI 1640 medium/10% pooled normal human serum (Innovative research) (HBMEC) or DMEM/10% HS (293T cells and fibroblasts). After 4 hours of infection, the number of adherent cell-bacteria in the supernatant was determined by lysis of HBMEC with 1% saponin for 15 min and subsequent determination of colony-forming units (cfu) by plating appropriate dilutions of the lysates of blood agar (bioMérieux, France). Intracellular bacteria were determined after 2 h of incubation with cell culture medium containing gentamicin (Biochrom, Berlin, Germany) at a concentration of 200 µg ml^−1^. The proportion of invasive bacteria was calculated as a ratio of the number of intracellular bacteria and the number of total cell-associated bacteria. All samples were tested in duplicate, and experiments were repeated at least three times. Bacterial viability was not affected either by saponin lysis or by addition of specific inhibitors as measured by colony plating. Effects of the inhibitors on cell viability were assessed by trypan exclusion method.

### siRNA Transfection

Validated cortactin siRNA (sc-35093) was synthesized by Santa Cruz Biotechnology, Santa Cruz, CA. A pool of 3 different twenty-one base sequences siRNA duplexes of the human cortactin gene (GenBank accession number NM_001184740.1, NM_005231.1, NM_1385565.2) was chosen. Validated FAK siRNA (sc-35353) was synthesized by Santa Cruz Biotechnology. Control nonsilencing siRNA (control siRNA-A from Santa Cruz) or cortactin siRNA or FAK siRNA, respectively, were transiently transfected into HBMEC growing in HBMEC medium using 3 µl of HiPerfect Transfection Reagent (Qiagen) according to the manufacturer’s instructions. Protein knockdown efficiencies by siRNA transfection were verified by immunoblotting after 72 h of transfection.

### DNA Expression Plasmids and Transfection of Cells

Expression constructs encoding for HA-tagged wildtype mouse FAK, HA-tagged kinase-inactive mouse FAK (FAK K454M), FAK Y397F and HA-tagged FRNK were described previously [69], [70]. FLAG-tagged cortactin wildtype (WT), cortactin W22A and cortactin W525K were kindly provided by Scott Weed (University of Colorado, Denver, CO). pcDNA3.1 was purchased from Invitrogen (Carlsbad, CA) and used as a negative control in transfection experiments. 293T cells were transfected with the calcium phosphate co-precipitation protocol using 1 µg plasmid DNA. In brief, 293T cells were seeded onto 24 well tissue culture plates and were grown to semi-confluence. Cells were then incubated with 1 µg plasmid DNA in 2xBBS (50 mM N,N-bis-(2-Hydroxyethyl)-2-aminoethanesulfonic acid (BES, Calbiochem, La Jolla, CA, USA), 180 mM NaCl, 1,5 mM Na_2_HPO_4_×H_2_O) and 220 mM CaCl_2_. After 24 h incubation, DMEM medium was replaced. Transfection efficiencies varied between 70 to 80% and were monitored by parallel transfection with a green fluorescent actin expressing construct (pTagGFP2-actin vector, Evrogen, Moscow, Russia) and quantification of fluorescent cells. Cells were used in infection experiments 48 h after transfection.

### Western Blots Analysis

FAK and cortactin expression levels were examined in cell extracts prepared in parallel to cells used for invasion assays. 4 h post infection, cells were washed three times in ice-cold phosphate buffered saline (PBS, Biochrome AG, Berlin, Germany) and lysed in 2xSDS sample buffer (SDS, Sigma Chemie GmbH, Steinheim, Germany). Samples were boiled, and proteins were separated by SDS-Page. After electrotransfer onto nitrocellulose (Schleicher and Schuell, Dassel, Germany), membranes were blocked in PBS/0,1% Tween containing 6% dry milk (Bio-Rad, Munich, Germany). Membranes were probed with α-HA (1∶1000), α-p-Tyr-100 (1∶1000), and α-cortactin (1∶2000) antibodies at 4°C overnight. After washing three times with PBS/Tween, membranes were incubated with horseradish peroxidase (HRP)-conjugated secondary antibodies (α-mouse 1∶10000; α-rabbit 1∶5000 in 6% dry milk/PBS/Tween) for 1 h. Immunoreactivity was detected using the ECL (enhanced chemiluminescence) reagent (Pierce, Rockford, IL, USA).

### Quantification of Band Intensity

To quantify the bands we applied ImageJ software band analysis (http://rsb.info.nih.gov./nih-ij). The area under the curve (AUC) of the specific signal was corrected for the AUC of the loading control (β-actin). The value for the p80/85 signal of the control cells was set as 1 and the value of p80/85 at the other time points were recalculated correspondingly to allow ratio comparisons.

### Immunoprecipitation

At indicated time points, infected cells were washed twice with ice-cold PBS and lysed in modified RIPA2 buffer (50 mM Tris-HCl, 150 mM NaCl, 5 mM EDTA, 1% Triton X®-100, 0.1% SDS, 24 mM sodium deoxycholate, 50 mM NaF, 0.2 mM sodium orthovanadate, 1 mM 1,10-phenantroline monohydrate, and protease inhibitor cocktail tablet (Roche Diagnostics, Mannheim, Germany)). After mechanically destruction of cells and incubation on ice for 30 min, the lysates were spun down and the supernatant was added with 1 µg IgG rabbit and 20 µl G plus agarose (both from Santa Cruz Biotechnology, Santa Cruz, CA, USA). The probes were again spun down after 30 min and equal amounts of supernatant were incubated with an α-cortactin antibody at a final dilution at 1∶1000 and 30 min later additionally with 20 µl G plus agarose. Probes were incubated overnight, spun down and the pellets were washed two times in PBS containing protease inhibitor cocktail tablet, 0.2 mM sodium orthovanadate and 1 mM 1,10-phenantroline monohydrate. For Western blot analysis, the precipitates were resuspended in reducing 2× SDS sample buffer and analyzed as described above. All incubations were performed at 4°C. Protein content in the supernatants was quantified by using the Lowry method with BSA as the standard.

### Fluorescence Staining and Microscopy

For determination of extra- and intracellular bacteria a double cycle antibody staining protocol was used [8]. Fibroblasts were seeded on glass coverslips in 24-well plates and grown to semi-confluence. Cells were infected with Neisseria at an MOI of 10. Cells were rinsed gently with PBS to remove extracellular non-adherent bacteria and fixed with 3.7% paraformaldehyde/PBS for 20 min at room temperature. Paraformaldehyde-fixed cells were washed three times with PBS and extracellular bacteria were stained for 20 min with suitable dilution of polyclonal rabbit-α- meningococcal outer membrane protein (OMP) antiserum (1∶100, in house production) in blocking buffer (1xPBS/2% FCS/1.5% BSA) at room temperature. Samples were washed twice with PBS, blocked again and incubated for 20 min with AMCA-conjugated goat-α-rabbit IgG (1∶200, Molecular Probes/Invitrogen) at room temperature. After two washes with PBS, samples were incubated with 0.5% Triton® X-100 to permeabilize cellular membranes. Cells were again incubated with polyclonal rabbit-α-meningococcal OMP antiserum in blocking buffer for 20 min at room temperature to stain intracellular located bacteria, washed two times with PBS, and stained for 20 min with TRITC-conjugated goat-α-rabbit IgG (1∶200, DIANOVA). After two washes, cells were stained with Alexa Fluor® 488 phalloidin (1∶50, Molecular Probes) for 20 min at room temperature. After further three washes with PBS, samples were mounted on slides in quick-hardening mounting medium (Fluka, Steinheim, Germany).

For detecting recruitment of cortactin, 293T cells were infected with FITC-labeled bacteria [71] and fixed cells were probed with goat α-rabbit cortactin antibody (1∶50) followed by incubation with secondary TRITC-conjugated antibody (1∶200) in blocking buffer for 60 min at room temperature. For a close-up view of the increase of cortactin and actin adjacent to attached bacteria and to prove that cells are not apoptotic, cells were additionally incubated with Alexa Fluor® 488 phalloidin, 4′,6-diamidino-2-phenylindole (DAPI, 1∶50000, Molecular Probes/Invitrogen), MitoTracker (1∶200, Molecular Probes/Invitrogen). Bacteria were either FITC-labeled or were stained with mouse-α-meningococcal antiserum and the secondary antibodies TRITC goat α-mouse or Alexa Fluor® 594 goat α-mouse (both 1∶200, Molecular Probes/Invitrogen).

For immunofluorescence analyses of actin accumulation HBMEC were infected with *N. meningitidis* for 4 h. Infected cells were fixed and labeled with Alexa Fluor® 488 phalloidin and bacteria were immunostained with polyclonal rabbit-α-meningococcal OMP antiserum and secondary TRITC-labeled goat α-rabbit IgG.

All incubations were performed in a wet chamber at room temperature. Fluorescence microscopy was performed using the Zeiss Axio Imager.Z1 fluorescence microscope (Zeiss, Heidelberg, Germany) or the Keyence BZ-9000 microscope (Keyence, Neu-Isenburg, Germany) and a CFI Plan Apo VC60xH oil objective. Images were photographed using either AxioCam digital Camera and AxioVision software or the BZ-II Analyzer software and documented using Adobe Photoshop CS.

### Statistical Analysis

Statistical differences between groups were calculated using the Student’s unpaired *t*-test (two-tailed) using Excel. *P*-values ≤0.05 were considered significant, *P*-values ≤0.01 were considered highly significant.

## Supporting Information

Figure S1 293T cells were again transiently transfected with the mutant form cortactin W22A (mutant form of cortactin in the NTA domain, which is no longer able to bind the Arp2/3 complex) or cortactin W525K (point mutation in the SH3 domain, mutant form impaired in the ability to bind dynamin-2), respectively, followed by an α-cortactin IP. Immunoprecipitates were analyzed with an α-Arp2/3 or α-dynamin antibody and revealed that dynamin did not bind to the cortactin W525K construct (Fig. S1B). The Arp2/3 complex binding was significantly less compared to cortactin W22A compared to cortactin W525K construct as demonstrated by densitometric analysis (Fig. S1A).
(TIF)
