# Supplementary figures and images for: Cell Invasion by Neisseria meningitidis Requires a Functional Interplay between the Focal Adhesion Kinase, Src and Cortactin
Source: PLoS One. 2012 Jun 29;7(6):e39613. doi: 10.1371/journal.pone.0039613 (PMC3387252; doi:10.1371/journal.pone.0039613)

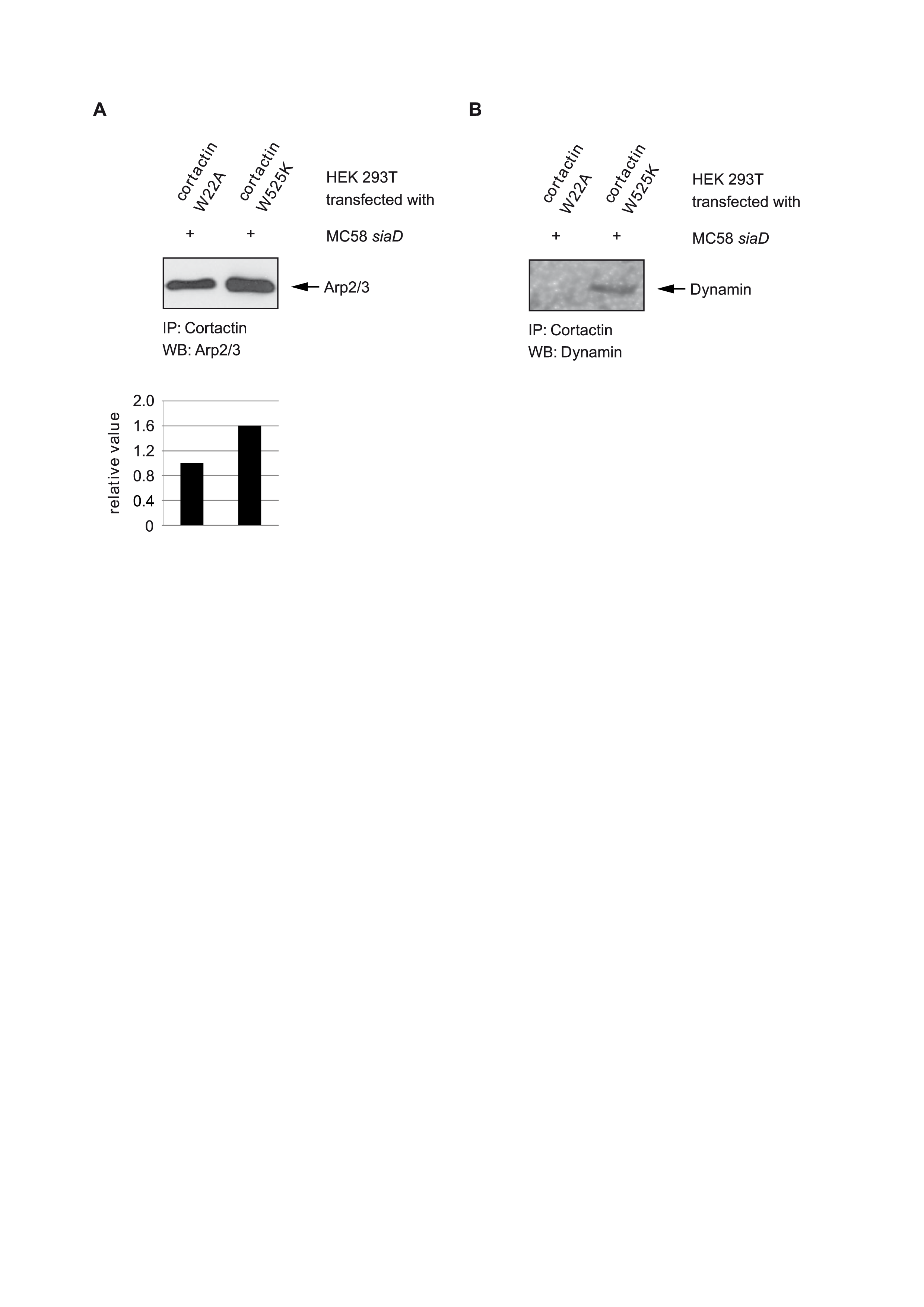

Supplement: Figure S1 — 293T cells were again transiently transfected with the mutant form cortactin W22A (mutant form of cortactin in the NTA domain, which is no longer able to bind the Arp2/3 complex) or cortactin W525K (point mutation in the SH3 domain, mutant form impaired in the ability to bind dynamin-2), respectively, followed by an α-cortactin IP. Immunoprecipitates were analyzed with an α-Arp2/3 or α-dynamin antibody and revealed that dynamin did not bind to the cortactin W525K construct (Fig. S1B). The Arp2/3 complex binding was significantly less compared to cortactin W22A compared to cortactin W525K construct as demonstrated by densitometric analysis (Fig. S1A). (TIF) [file pone.0039613.s001.tif]
